# Facial Skin Quality Improvement After Treatment With CPM‐HA20G: Clinical Experience in Korea

**DOI:** 10.1111/jocd.16795

**Published:** 2025-01-22

**Authors:** Je‐Young Park, ChoonShik Youn, Chanwoo Lee, Kyou Chae Lee, Heawon Shin, Kkot Bora Yeom, Juhyuk Park, Sungkyu Jung, Joo Ha Kim, Wonkyu Hong

**Affiliations:** ^1^ Apkoo‐Jung Oracle Dermatology Clinic Seoul Republic of Korea; ^2^ Yemiwon Dermatologic Clinic Seoul Republic of Korea; ^3^ The CleanN Dermatologic Clinic Cheonan Republic of Korea; ^4^ Hwanggeum Dermatology Clinic Daegu Republic of Korea; ^5^ Oracle Dermatology Clinic Seoul Republic of Korea; ^6^ Seoul One Dermatology Clinic Seoul Republic of Korea; ^7^ The Heal Dermatology Clinic Seoul Republic of Korea; ^8^ Doctors Dermatology Clinic Seoul Republic of Korea; ^9^ Story Dermatologic Clinic Seoul Republic of Korea; ^10^ Human Dermatology Clinic Incheon Republic of Korea; ^11^ Human Co. Ltd. Skin Clinical Trial Center Seoul Republic of Korea

**Keywords:** Asian, cohesive polydensified matrix, facial rejuvenation, filler, hyaluronic acid, skin quality

## Abstract

**Background:**

Intradermal injection of CPM‐HA20G, a low‐viscoelasticity hyaluronic acid (HA) dermal filler with glycerol, has been shown to be effective for facial rejuvenation in Caucasians, but research in Asians is limited.

**Aims:**

This study aimed to evaluate the effectiveness and safety of CPM‐HA20G in enhancing facial skin quality in Korean women using a protocol developed by local aesthetic experts.

**Patients/Methods:**

In this 24‐week prospective, single‐arm, open‐label study, 20 women received CPM‐HA20G injections in the immediate subdermal layer on the anterior cheek (1 mL per side; total 2 mL) in three sessions every 4 weeks. Evaluations included biophysical assessments covering the four emergent perceptual categories (EPCs) for skin quality and subjective assessment using the Global Aesthetic Improvement Scale.

**Results:**

Significant improvements in skin glow (skin gloss, epidermal hydration), skin firmness (dermal elasticity, dermal hydration), skin surface evenness (average pore volume, pore area, pore density, pore count, maximum pore depth, total pore volume, skin roughness, sebum secretion, and skin depression volume), skin tone evenness (skin color brightness, skin redness), and transepidermal water loss were observed at Week 12 and Week 24 following the first injection with CPM‐HA20G. Most subjects and investigators reported improvements in overall aesthetic appearance with the treatment, with a 100% improvement rating from both groups at Week 12. No serious adverse reactions were observed.

**Conclusions:**

Our study provides real‐world insights into the effectiveness and safety of CPM‐HA20G in improving facial skin quality in an Asian population, evaluated through both objective and subjective assessments.

## 1 Introduction

Facial skin quality profoundly impacts perceived attractiveness and youthfulness, motivating many individuals to seek aesthetic procedures to enhance their appearance [1, 2]. A panel of global aesthetic experts identified four attributes, known as emergent perceptual categories (EPCs), that describe good skin quality across different ethnicities: (i) skin glow, (ii) skin firmness, (iii) skin surface evenness, and (iv) skin tone evenness [1]. The perception of good skin quality is shaped by several attributes or EPCs, which are interconnected and can influence each other [1, 2]. For example, both skin glow and skin surface evenness can affect the perception of skin tone, while skin hydration can impact both skin surface evenness and skin elasticity [2]. Therefore, treatments that address multiple EPCs are desirable for a holistic and balanced outcome [1, 2].

Hyaluronic acid (HA) dermal fillers are increasingly used for facial rejuvenation [3, 4, 5]. They are minimally invasive, have a short recovery time and minimal side effects when administered properly by skilled practitioners, and can treat a range of skin concerns [2, 3, 4, 5]. A systematic review of 13 studies showed that intradermal HA filler injections improved skin hydration, firmness, brightness, texture, radiance, and elasticity [4], all of which are crucial for enhancing overall skin quality. Several HA fillers are available, distinguished by crosslinking technologies: non‐cohesive biphasic fillers, monophasic monodensified fillers, and cohesive monophasic polydensified matrix (CPM) gels [6, 7]. CPM‐HA is manufactured with Dynamic Cross‐Linking Technology (DCLT), which utilizes two cycles of crosslinking with butanediol diglycidyl ether (BDDE) to produce a gel with zones of greater and lesser density, facilitating homogeneous tissue integration and uniform enhancement across the treated areas [6, 7]. CPM‐HA20G (Belotero Revive, Merz Pharmaceuticals GmbH, Frankfurt, Germany) is a low‐viscoelasticity HA filler manufactured using the DCLT with glycerol incorporated [8, 9, 10]. It is suited for superficial injections in facial rejuvenation [8, 9]. Glycerol is a humectant known for its ability to enhance skin hydration, improve skin mechanical properties, reduce transepidermal water loss (TEWL), inhibit the stratum corneum lipid phase transition, provide anti‐irritant and anti‐inflammatory effects, accelerate wound healing, and exhibit antimicrobial actions [11, 12].

In a previous open‐label post‐marketing clinical study, CPM‐HA20G (administered in three sessions every 4 weeks) was evaluated in women showing signs of facial aging [8]. Improvements in skin elasticity, firmness, tone, radiance, hydration, fatigue, roughness, and redness were observed for up to 36 weeks after treatment. The study reported a favorable safety profile, with both subjects and investigators expressing high satisfaction with the outcomes [8]. A multicenter randomized controlled clinical study evaluated single and multiple doses of CPM‐HA20G (three sessions 4 weeks apart) in women with early‐onset photodamaged facial skin [9]. The study demonstrated enhanced skin hydration in subjects with dry or very dry skin for up to 9 months after the last injection in both groups [9]. Subject‐reported outcomes indicated that most subjects benefited from the treatment, reporting improved skin hydration, softness, tone, refreshing effect, elasticity, and smoothness. All adverse events (AEs) were mild to moderate and transient [9].

Available published data on CPM‐HA20G are mostly from studies on Caucasian populations [8, 9]. However, differences in skin characteristics between Asians and Caucasians, such as skin thickness, elasticity, and collagen distribution, can impact treatment effectiveness [13]. For instance, Asians tend to develop wrinkles later but experience more pigmentation issues, have higher sebum secretion, and have a thicker dermis compared with Caucasians [13, 14]. Additionally, a survey of 153 aesthetic practitioners in the Asia‐Pacific region identified skin quality concerns prevalent among Asians, including uneven skin tone, skin surface unevenness, skin laxity, sebaceous gland hyperactivity, enlarged pores, and dull and dry skin [2]. The impact of these unique attributes of Asian skin and aesthetic concerns on the efficacy and safety of HA fillers remains unclear and has not yet been investigated. Conducting studies on Asians will provide insights into the aesthetic outcomes and potential adverse effects of CPM‐HA20G in this population.

This study aimed to evaluate the effectiveness and safety of CPM‐HA20G in Korean women using a protocol developed by local aesthetic experts to improve facial skin quality. To reflect real‐world clinical experiences and provide practical insights into treatment outcomes, the study employed a single‐arm, open‐label design. A comparator was not included as the injection protocol was specifically tailored for CPM‐HA20G. Assessments included objective biophysical evaluation of all four EPCs for skin quality and subjective evaluation by both investigators and subjects on overall aesthetic appearance over 24 weeks.

## 2 Methods

### 2.1 Study Design, Study Population, and Treatment

This prospective, single‐arm, open‐label study was conducted at a centralized research center and the practicing institutions of 10 investigators in Korea between February and August 2023. The study received approval from a central institutional review board (IRB number: HM‐IRB‐P22‐0362; approval date: Jan 7, 2023), and all subjects provided written informed consent before the study commenced. Additionally, subjects gave written informed consent for the publication of their photographs. The study was conducted in compliance with good clinical practice guidelines and applicable local regulations.

**FIGURE 1:**
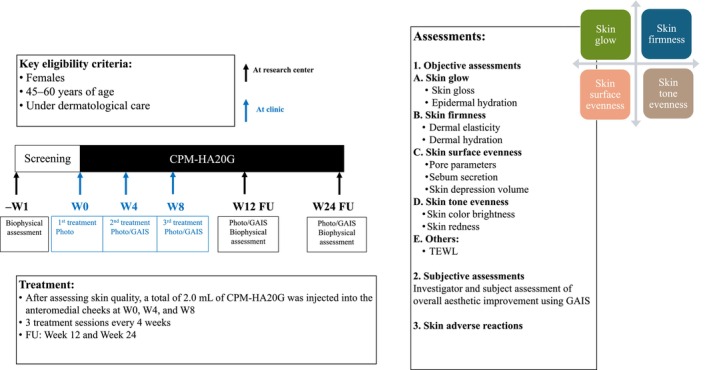
Study design. D, day; FU, follow‐up; GAIS, Global Aesthetic Improvement Scale; TEWL, transepidermal water loss; W, Week.

Healthy women aged 45 to 60 years desiring improvement in skin quality were eligible for the study (refer to Appendix S1 for the complete list of eligibility criteria). The study consisted of six visits: screening was conducted at Visit 1 (Week −1), and the study treatments were administered at Visit 2 (Week 0), and every 4 weeks after that at Visit 3 (Week 4) and at Visit 4 (Week 8). The follow‐up assessments were performed at Visit 5 (Week 12) and Visit 6 (Week 24) (Figure 1).

**FIGURE 2:**
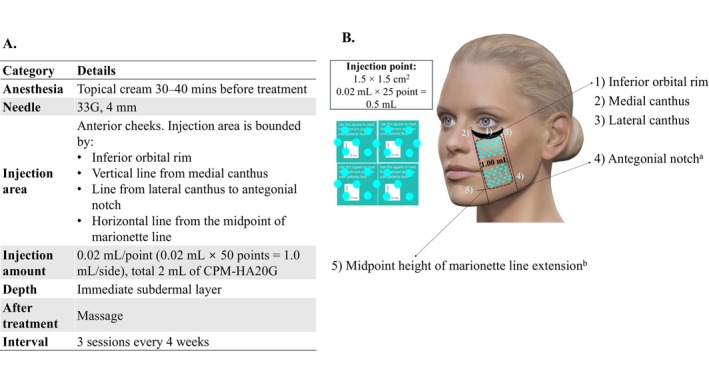
Study treatment. (A) protocol; (B) facial mapping. ^a^Assumed to be at the anterior border of the masseter in cases where it is difficult to palpate the mandibular notch. ^b^Avoid injection into jowls.

CPM‐HA20G is a CPM‐HA filler containing cross‐linked sodium hyaluronate gel (20 mg/mL) and glycerol (17.5 mg/mL). The authors developed the injection protocol for CPM‐HA20G for patients in Korea, based on published scientific evidence related to CPM‐HA20G [8, 9, 10] and their collective clinical experience in treating patients within the local population. The resulting treatment protocol is illustrated in Figure 2 and was implemented in all participating subjects. After assessing skin quality, a total of 2 mL of CPM‐HA20G was injected into the immediate subdermal layer of the anterior cheeks (1.0 mL per side; 0.02 mL per injection point, distributed across 50 points per side). Injections were administered over three sessions every 4 weeks (Figure 2).

### 2.2 Study Assessments

Evaluation of facial skin quality included objective biophysical assessments of the four EPCs for skin quality: (i) skin glow (skin gloss, epidermal hydration); (ii) skin firmness (dermal elasticity, dermal hydration); (iii) skin surface evenness (pore parameters, sebum secretion, and skin depression volume); (iv) skin tone evenness (skin color brightness, skin redness); and other attributes (TEWL) before treatment and at Week 12 and Week 24 (Figure 1). The biophysical parameters of skin quality were measured using corresponding devices (Table 1). We have divided skin hydration into epidermal hydration (assessed by Epsilon E100 in *ε*) and dermal hydration (measured by MoisturemeterD in tissue dielectric constant [TDC] [15]). In line with the four EPCs defined in the global consensus on skin quality [1], we considered that dermal hydration impacts skin firmness, while epidermal hydration, which affects the skin's superficial layers, primarily influences skin gloss.

**TABLE 1:** Biophysical parameters of skin quality measured in the study.

Parameters	Measurement devices	Unit of measurement and interpretation
Skin gloss	SkinGlossMeter (Delfin Technologies Ltd., Finland)	a.u. A higher value indicates greater skin glossiness
Epidermal hydration	Epsilon E100 (Biox Systems Ltd., England)	*ε* A brighter image indicates a higher value and a higher moisture content
Dermal elasticity (R2)	Cutometer MPA580 (Courage + Khazaka GmbH, Germany)	mm A higher value denotes more elastic skin
Dermal hydration	MoisturemeterD (Delfin Technologies Ltd., Finland)	TDC TDC measures conductivity at specific depths, and a higher value corresponds to a higher moisture content and reflects dermal hydration
Average pore volume	Antera 3D (Miravex Ltd., Ireland)	mm^3^ A lower value indicates reduced pore volume
Average pore area		mm^2^ A lower value denotes a reduced pore area
Pore density		No. of pores/cm^2^ A lower value denotes a lower pore density
Pore count		No. of pores A lower value indicates fewer pores
Maximum pore depth		mm A lower value indicates an improvement in maximum pore depth
Total pore volume		mm^3^ A lower value corresponds to reduced total pore volume
Skin roughness (Ra)		μm A lower value denotes reduced skin roughness
Skin redness		a* A lower value indicates reduced skin redness
Skin depression volume		mm^3^ Reduced skin depression volume indicates an improvement in the overall depth of wrinkles
Sebum secretion	Sebumeter SM815 (Courage+Khazaka GmbH, Germany)	μg/cm^2^ A lower value denotes reduced sebum secretion
Skin brightness	VISIA‐7 system (Canfield Scientific Inc., USA)	L* A higher value indicates a brighter skin
TEWL	VapoMeter (Delfin Technologies Ltd., Finland)	g/m^2^h A lower value denotes better skin barrier function

Abbreviations: Ra, average roughness; TDC, tissue dielectric constant; TEWL, transepidermal water loss.

Subjective assessment included the Global Aesthetic Improvement Scale (GAIS), which was completed by the investigators (investigator‐GAIS) and subjects (subject‐GAIS) at all post‐treatment visits (Weeks 4, 8, 12, and 24). GAIS uses a 5‐point scale (1 = very much improved to 5 = worse) to assess changes in overall aesthetic appearance compared with baseline. The investigators used 2‐dimensional photos taken before treatment and at each follow‐up visit for assessment (VISIA‐7 system, Canfield Scientific Inc., USA).

Adverse reactions were recorded throughout the study by the research personnel at the study center and through subject self‐reporting via a questionnaire. Participants received written post‐treatment instructions in the clinic following each CPM‐HA20G injection.

### 2.3 Statistical Analyses

The results were expressed as the mean and standard deviation (SD) for continuous variables and as frequency and percentage for categorical variables. The normality of the data was verified using the Shapiro–Wilk test. For comparing values between specific time points and before treatment, the paired *t*‐test was employed when the assumption of normality was met; otherwise, the Wilcoxon signed‐rank test was applied. The corresponding 95% CI for the percent change was calculated using the *t*‐distribution based on the mean change between pre‐treatment and the specific time point, and the corresponding standard error. All statistical analyses were performed using SPSS Version 26 (SPSS Inc., Chicago, IL, USA). *p* < 0.05 were considered statistically significant.

## 3 Results

### 3.1 Subject Demographic and Characteristics

Of the 22 subjects enrolled, two did not attend the visit at Week 24. The remaining 20 subjects completed the study and were included in the analysis. The mean age was 54.4 (SD 4.4) years. Before treatment, most subjects had dry skin (75%), normal skin texture (75%), and normal skin tone (70%), with all subjects being nonsmokers (100%) (Table 2).

**TABLE 2:** Subject demographics and characteristics.

	Overall (*N* = 20)
Age, years	
Mean (SD)	54.4 (4.4)
Gender, *n* (%)	
Female	20 (100.0)
Facial skin type, *n* (%)	
Dry	15 (75.0)
Normal	1 (5.0)
Oily	0 (0.0)
Combination	4 (20.0)
Skin color tone, *n* (%)	
Bright	1 (5.0)
Normal	14 (70.0)
Dark	5 (25.0)
Skin texture, *n* (%)	
Smooth	3 (15.0)
Normal	15 (75.0)
Rough	2 (10.0)
Skin moisture, *n* (%)	
Moist	1 (5.0)
Normal	6 (30.0)
Dry	13 (65.0)
Smoking history	
Nonsmoker	20 (100.0)

*Note:* Percentages were calculated based on the number of subjects (*N*).

Abbreviations: *N*, number of subjects; *n*, number of subjects in the specified category; SD, standard deviation.

### 3.2 Objective Assessments of Facial Skin Quality

**FIGURE 3:**
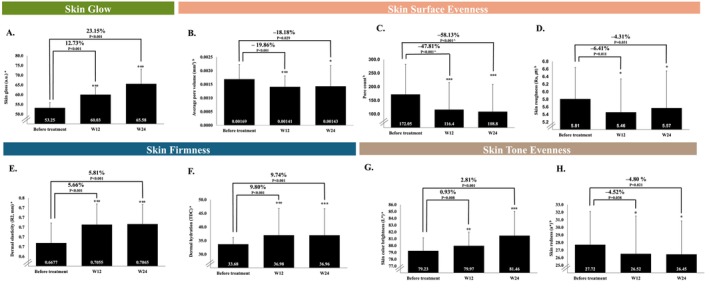
Effects on (A) skin gloss, (B) average pore volume, (C) pore count, (D) skin roughness, (E) dermal elasticity, (F) dermal hydration, (G) skin color brightness, and (H) skin redness after treatment with CPM‐HA20G at Week 12 and Week 24. *N* = 20 for all parameters at all time points. ^a^The rate of change (%) was calculated using the formula: (values after treatment–values before treatment)/values before treatment ×100. ^b^The rate of change (%) was calculated using the formula: (values before treatment–values after treatment)/values after treatment ×100. Paired *t*‐tests were conducted to obtain the *p*‐value unless marked with ^, which indicates the use of the Wilcoxon signed‐rank test. Statistical significance was determined as *p* < 0.05, with a comparison made to the value before treatment. ***p < 0.001; **p < 0.01; *p < 0.05. Results presented are mean (SD) unless otherwise stated. Percentage represents percent change. Ra, average roughness; W, Week.

**FIGURE 4:**
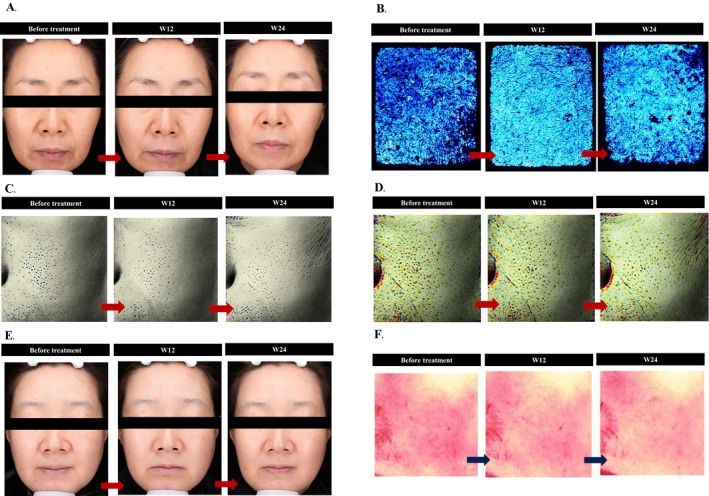
Representative images depicting improvements in skin quality after treatment with CPM‐HA20G at Week 12 and Week 24. (A) skin glow; (B) epidermal hydration; (C) skin pore; (D) skin roughness; (E) skin color brightness; (F) skin redness. W, Week.

The objective biophysical assessment results are presented in Table 3. All outcomes showed significant improvement at both Week 12 and Week 24 compared with before treatment (*p* < 0.05). Key results are illustrated in Figure 3. Figure 4 shows representative images depicting improvements in skin quality (skin glow, epidermal hydration, skin pores, skin roughness, skin color brightness, and skin redness), encompassing all four EPCs of skin quality, after treatment with CPM‐HA20G.

**TABLE 3:** Effects on skin glow, skin firmness, skin surface evenness, skin tone evenness, and other outcomes after treatment with CPM‐HA20G.

Parameters	Before treatment *N* = 20	Week 12 *N* = 20				Week 24 *N* = 20			
	Mean (SD)	Mean (SD)	Percent change (%)	95% CI	*p*‐values	Mean (SD)	Percent change (%)	95% CI	*p*
Skin glow									
Skin gloss (a.u.)^a^	53.25 (2.71)	60.03 (3.56)	12.73	8.97; 16.49	< 0.001	65.58 (7.53)	23.15	16.43; 29.88	< 0.001
Epidermal hydration (ε)^a^	26.84 (7.13)	39.22 (7.28)	46.13	29.13; 63.12	< 0.001	34.41 (7.61)	28.20	10.81; 45.59	0.002
Skin firmness									
Dermal elasticity, *R* ^2^ (mm)^a^	0.6677 (0.0410)	0.7055 (0.0422)	5.66	1.72; 9.61	< 0.001	0.7065 (0.0404)	5.81	1.95; 9.67	< 0.001
Dermal hydration (TDC)^a^	33.68 (2.48)	36.98 (2.31)	9.80	5.29; 14.30	< 0.001	36.96 (2.54)	9.74	5.02; 14.46	< 0.001
Skin surface evenness									
Average pore volume (mm^3^)^b^	0.00169 (0.00054)	0.00141 (0.00040)	−19.86	−41.13; 1.41	< 0.001^	0.00143 (0.00076)	−18.18	−47.29; 10.93	0.029^
Average pore area (mm^2^)^b^	0.1186 (0.0420)	0.1051 (0.0354)	−12.84	−36.24; 10.55	0.005	0.1126 (0.0414)	−5.33	−28.77; 18.11	< 0.001
Pore density (no. of pores/cm^2^)^b^	10.99 (6.88)	7.43(6.23)	−47.91	−103.84; 8.01	< 0.001	9.74 (6.00)	−12.83	−54.78; 29.12	0.040
Pore count^b^	172.05 (110.35)	116.40 (99.48)	−47.81	−104.94; 9.32	< 0.001^	108.80 (100.08)	−58.13	−119.42; 3.15	< 0.001^
Maximum pore depth (mm)^b^	0.0249 (0.0060)	0.0211 (0.0054)	−18.01	−35.13; −0.89	< 0.001	0.0221 (0.0055)	−12.67	−29.15; 3.82	0.006
Total pore volume (mm^3^)^b^	0.3412 (0.3518)	0.2006 (0.2468)	−70.09	−165.98; 25.80	< 0.001	0.2236 (0.2361)	−52.59	−137.41; 32.22	0.017^
Skin roughness (Ra, μm)^b^	5.81 (0.83)	5.46 (0.88)	−6.41	−16.33; 3.51	0.011	5.57 (0.98)	−4.31	−14.63; 6.01	0.031
Sebum secretion (μg/cm^2^)^b^	40.45 (5.52)	23.65 (5.43)	−71.04	−85.69; −56.38	< 0.001	25.05 (7.82)	−61.48	−78.58; −44.37	< 0.001
Skin depression volume (mm^3^)^b^	1.067 (0.807)	0.924 (0.732)	−15.48	−68.25; 37.30	0.032	0.920 (0.831)	−15.98	−72.33; 40.38	0.036
Skin tone evenness									
Skin color brightness (L*)^a^	79.23 (1.92)	79.97 (2.00)	0.93	−0.63; 2.50	0.008	81.46 (3.57)	2.81	0.52; 5.10	< 0.001
Skin redness (a*)^b^	27.72 (4.37)	26.52 (4.98)	−4.52	−15.71; 6.66	0.038	26.45 (4.41)	−4.80	−15.31; 5.71	0.021
Others									
TEWL (g/m^2^h)^b^	12.65 (2.05)	10.76 (1.64)	−17.57	−28.48; −6.63	< 0.001	9.38 (1.67)	−34.86	−47.48; −22.24	< 0.001

*Note:* Paired *t*‐tests were conducted to obtain the *p*‐value, unless marked with ^, which indicates the use of the Wilcoxon signed‐rank test. Statistical significance was determined as *p* < 0.05, with a comparison made to the value before treatment. The 95% CI for the percent change was calculated using the *t*‐distribution based on the mean change between pre‐treatment and the specific time point, and the corresponding standard error. The colours are designed to match the relevant EPCs in Figure 1 and Figure 3.

Abbreviations: CI, confidence interval; Ra, average roughness; TDC, tissue dielectric constant; TEWL, transepidermal water loss.

^a^ The percent change was calculated using the formula: (values after treatment–values before treatment)/values before treatment ×100.

^b^ The percent change was calculated using the formula: (values before treatment–values after treatment)/values after treatment ×100.

#### 3.2.1 Skin Glow Outcomes

The mean skin gloss improved at Week 12 and Week 24 compared with before treatment, with a corresponding percent change of 12.73% (95% confidence interval [CI]: 8.97% to 16.49%; *p* < 0.001) and 23.15% (95% CI: 16.43% to 29.88%; *p* < 0.001), respectively (Figure 3A). The mean skin epidermal hydration improved at Week 12 and Week 24 compared with before treatment. The corresponding percent change was 46.13% (95% CI: 29.13% to 63.12%; *p* < 0.001) and 28.20% (95% CI: 10.81% to 45.59%; *p* < 0.001), respectively. The improvements in skin gloss and hydration enhanced the perception of a healthier, more radiant skin glow.

#### 3.2.2 Skin Surface Evenness Outcomes

Skin surface evenness metrics showed consistent improvements at Weeks 12 and 24 compared with before treatment, as reflected in the mean percent change values. The average pore volume improved at Week 12 and Week 24 compared with before treatment, with a corresponding percent change of −19.86% (95% CI: −41.13% to 1.41%; *p* < 0.001) and −18.18% (95% CI: −47.29% to 10.93%; *p* = 0.029), respectively (Figure 3B). Pore area improved at Week 12 and Week 24 compared with before treatment. The corresponding percent change was −12.84% (95% CI: −36.24% to 10.55%; *p* = 0.005) and − 5.33% (95% CI: −28.77% to 18.11%; *p* < 0.001), respectively. Pore density improved at Week 12 and Week 24 compared with before treatment, with a corresponding percent change of −47.91% (95% CI: −103.84% to 8.01%; *p* < 0.001) and −12.83% (95% CI: −54.78% to 29.12%; *p* = 0.040), respectively. Pore count improved at Week 12 and Week 24 compared with before treatment, with a corresponding percent change of −47.81% (95% CI: −104.94% to 9.32%; *p* < 0.001) and −58.13% (95% CI: −119.42% to 3.15%; *p* < 0.001), respectively (Figure 3C). Maximum pore depth improved at Week 12 and Week 24 compared with before treatment, with a corresponding percent change of −18.01% (95% CI: −35.13% to −0.89%; *p* < 0.001) and −12.67% (95% CI: −29.15% to 3.82%; *p* = 0.006), respectively. Total pore volume improved at Week 12 and Week 24 compared with before treatment, with a corresponding percent change of −70.09% (95% CI: −165.98% to 25.80%; *p* < 0.001) and −52.59% (95% CI: −137.41% to 32.22%; *p* = 0.017), respectively. Skin roughness improved at Week 12 and Week 24 compared with before treatment, with a corresponding percent change of −6.41% (95% CI: −16.33% to 3.51%; *p* = 0.011) and −4.31% (95% CI: −14.63% to 6.01%; *p* = 0.031), respectively (Figure 3D). Sebum secretion improved at Week 12 and Week 24 compared with before treatment. The corresponding percent change was −71.04% (95% CI: −85.69% to −56.38%; *p* < 0.001) and −61.48% (95% CI: −78.58% to −44.37%; *p* < 0.001), respectively. Skin depression volume improved at Week 12 and Week 24 compared with before treatment, with a corresponding percent change of −15.48% (95% CI: −68.25% to 37.30%; *p* = 0.032) and −15.98% (95% CI: −72.33% to 40.38%; *p* = 0.036), respectively. The improvements in pore parameters, skin roughness, sebum secretion, and skin depression volume led to a more even and refined skin surface.

#### 3.2.3 Skin Firmness Outcomes

The mean dermal elasticity improved at Week 12 and Week 24 compared with before treatment. The corresponding percent change was 5.66% (95% CI: 1.72% to 9.61%; *p* < 0.001) and 5.81% (95% CI: 1.95% to 9.67%; *p* < 0.001), respectively (Figure 3E). The mean dermal hydration improved at Week 12 and Week 24 compared with before treatment, with a corresponding percent change of 9.80% (95% CI: 5.29% to 14.30%; *p* < 0.001) and 9.74% (95% CI: 5.02% to 14.46%; *p* < 0.001), respectively (Figure 3F). The improvements in dermal elasticity and hydration resulted in more supple and firmer skin.

#### 3.2.4 Skin Tone Evenness Outcomes

The mean skin color brightness improved at Week 12 and Week 24 compared with before treatment. The corresponding percent change was 0.93% (95% CI: −0.63% to 2.50%; *p* = 0.008) and 2.81% (95% CI: 0.52% to 5.10%; *p* < 0.001), respectively (Figure 3G). The mean skin redness improved at Week 12 and Week 24 compared with before treatment. The corresponding percent change was −4.52% (95% CI: −15.71% to 6.66%; *p* = 0.038) and −4.80% (95% CI: −15.31% to 5.71%; *p* = 0.021) respectively (Figure 3H). The improvements in skin brightness and redness contributed to a more even and balanced skin tone.

#### 3.2.5 Other Biophysical Outcomes

The mean TEWL improved at Week 12 and Week 24 compared with before treatment, with a corresponding percent change of −17.57% (95% CI: −28.48% to −6.63%; *p* < 0.001) and −34.86% (95% CI: −47.48% to −22.24%; *p* < 0.001), respectively. The improvements in TEWL denote improvements in skin barrier function.

### 3.3 Subjective Assessment of Overall Aesthetic Appearance (GAIS)

**FIGURE 5:**
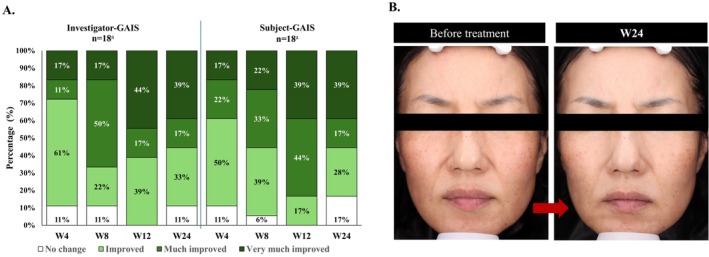
Overall aesthetic improvement as assessed by (A) investigator‐ and subject‐GAIS assessments from Week 4 to Week 24. (B) representative photographs of a subject who was rated ‘very much improved’ by both the investigator and the subject on the GAIS at Week 24 compared with Week 0. ^a^GAIS responses from two subjects were not recorded at Week 24 follow‐up and were not included in the analysis. GAIS, Global Aesthetic Improvement Scale; W, Week.

According to the investigator‐ and subject‐ GAIS assessments, a high proportion of subjects demonstrated improvements in overall aesthetic appearance from Week 0 at each post‐treatment visit. By Week 4, 89% of subjects showed improvement from Week 0 (investigator‐ and subject‐GAIS). The highest proportion of subjects with improvement was noted at Week 12 (investigator‐ and subject‐GAIS: 100% rated “improved”, “much improved”, or “very much improved”) and the proportion decreased slightly at Week 24 (investigator‐ and subject‐GAIS: 89% and 83%, respectively) (Figure 5). Representative figures illustrating the trends in outcomes across different time points are presented in Figure S1.

### 3.4 Adverse Reactions

Pain and discomfort at injection sites were tolerable with the use of topical anesthesia cream, and all subjects completed the full course of three treatment sessions. Common treatment‐related adverse reactions included erythema and bruising, which were mild and resolved without intervention. No serious adverse reactions, such as infection or necrosis, were observed.

## 4 Discussion

Our study assessed the effectiveness and safety of CPM‐HA20G in improving facial skin quality in an Asian population. Using a treatment protocol devised by local aesthetic experts, we observed enhancements in skin quality in Korean women following treatment with CPM‐HA20G. Objective biophysical assessments showed that skin quality parameters encompassing all EPCs significantly improved up to 24 weeks after treatment. Most investigators and subjects reported improvements in overall aesthetic appearance at all post‐treatment visits, with a 100% improved rating from both groups at Week 12. All adverse reactions observed throughout the study period were transient and mild.

The perception of good skin quality is influenced by several interconnected attributes or EPCs [1, 2]. Therefore, addressing these attributes is essential for achieving holistic facial rejuvenation [1, 2]. In the present study, objective biophysical assessments showed that multiple attributes improved after treatment with CPM‐HA20G, consistent with previous studies [8, 9]. Treatment with CPM‐HA20G showed significant improvements in skin gloss, epidermal hydration, dermal elasticity, dermal hydration, pore parameters, sebum secretion, skin brightness, skin redness, and TEWL, with marginal improvements in skin depression volume without producing a volumizing effect at Week 12 and Week 24 compared with before treatment. These improvements collectively led to a holistic and balanced enhancement in skin quality. These findings are particularly relevant for the Asian populations as the skin quality concerns identified as prevalent among Asians in an earlier survey [2] were effectively addressed with CPM‐HA20G treatment. This study's findings align with earlier research on CPM‐HA20G, which also demonstrated improvements in multiple skin quality attributes [8, 9]. Additionally, our study showed reduced sebum secretion and improved skin barrier function with CPM‐HA20G treatment, which have not been previously reported. This extends the existing published effects of CPM‐HA20G.

HA fillers have been shown to be effective for skin rejuvenation [4, 5, 8, 16, 17, 18, 19, 20, 21]. Upon superficial injection, HA with low viscoelasticity forms a condensed network with the extracellular matrix (ECM), which enhances hydration and activates fibroblasts. This leads to increased production of collagen, elastin, and HA, as well as the release of stromal cell‐derived factor 1 (SDF‐1), which aids in inhibiting skin pigmentation [2, 5, 22]. These processes collectively result in improved skin hydration and elasticity, reduced surface roughness, and enhanced skin tone [2, 5]. The effect of CPM‐HA20G in inhibiting sebum production may be attributed to HA's ability to downregulate lipid synthesis via CD44/RhoA signaling [23]. The improvements in pore parameters may be attributed to the stabilization of the ECM that supports fibroblasts, which in turn induces collagen production and improves skin elasticity [4, 24]. Downregulation of sebaceous gland activity and lipid synthesis may also play a role [23]. Furthermore, dermal hydration by HA can produce an expansion effect, leading to the perceived pore size reduction [24, 25].

The unique formulation of CPM‐HA20G, which includes glycerol as a humectant, may contribute to its rapid and enhanced effects. Glycerol has been demonstrated to moisturize, strengthen, and provide protective benefits to the skin [11, 12]. Physicochemical analysis of CPM‐HA20G demonstrated an immediate release of glycerol, which can penetrate into the human dermis in an ex vivo study [26]. A three‐dimensional analysis of a human skin model confirmed an early onset of skin hydration at the injection site [26]. The concerted effects of HA and glycerol likely contributed to overall improvements in skin quality, most notably for the deep and rapid enhancements in skin hydration and skin glow and maintenance of skin barrier integrity after treatment. In the present study, improvements in skin redness were more apparent in subjects with inflammation‐induced redness, such as rosacea and acne, than those with redness caused by aging or telangiectasia from sun exposure. This aligns with the known effects of HA and glycerol, which act as antioxidants to alleviate inflammatory conditions, potentially reducing skin redness [4, 5, 8, 11, 12, 16, 17, 18, 19, 20, 21]. Skin brightness was also significantly improved with CPM‐HA20G, likely due to the release of SDF‐1 and perceived brighter skin resulting from smoother skin and reduced redness [2, 5, 22].

As seen in previous studies [8, 9], we observed varying trends of improvement for different attributes during the follow‐up visits. In the present study, skin gloss, skin brightness, and TEWL continued to improve from Week 12 to Week 24. Improvements in dermal elasticity, dermal hydration, average pore volume, pore count, skin redness, and skin depression volume observed at Week 12 were maintained at Week 24. There was a slight decline in improvements for epidermal hydration, pore area, pore density, pore depth, total pore volume, roughness, and sebum secretion from Week 12 to Week 24. The effects of HA can be categorized into: (i) primary effects, arising from the initial volume of HA administered during treatment; and (ii) secondary maintenance effects, attributed to the enhanced microenvironment cultivated by HA [27, 28]. Following the third treatment, the cumulatively administered HA volume may have contributed to sustained improvements in attributes such as pore appearance and skin roughness up to Week 12. However, the absence of additional HA volume beyond this time point, coupled with the natural degradation of HA and glycerol over time, likely accounts for the slight reductions observed by Week 24 compared with the maximal improvements at Week 12, while substantial improvement from baseline is maintained. Meanwhile, improvements in skin glow, firmness, and tone are likely sustained by the secondary maintenance effects and enhanced dermal microenvironment. These trends underscore the multifaceted benefits of HA treatment. Touch‐up treatments are typically recommended every 6 months to maintain these effects. Further studies are needed to understand the observed trends for different attributes and the durability of the treatment effect with CPM‐HA20G. Nonetheless, the findings showed significant improvements in multiple attributes up to 24 weeks after treatment.

The results of GAIS assessments corroborated the positive findings from objective biophysical assessments, indicating enhanced facial skin quality and overall aesthetic appearance with CPM‐HA20G treatment for up to 24 weeks. According to both investigator‐ and subject‐GAIS, a high proportion of subjects showed improvements in overall aesthetic appearance at all post‐treatment visits. Improvements were evident as early as 4 weeks after a single treatment session, with the highest proportion of perceived improvements at Week 12 (100%), 4 weeks after the third treatment session. This highlights the rapid effect of CPM‐HA20G and that the most prominent improvement occurred after completing the treatment course. It is important to note that the trend and extent of improvements across time points varied among patients. While some demonstrated continuous or sustained improvements between Weeks 12 and 24, others exhibited maximal improvements in Week 12, underscoring the variability in individual responses.

No serious adverse reactions were reported in our study. Reported adverse reactions were mostly transient and mild injection site reactions, consistent with published data [9].

One of the strengths of the current study is the comprehensive assessment of both objective and subjective parameters, encompassing a broader range of assessments than previously published studies on CPM‐HA20G, which enhances the robustness of the study findings. Furthermore, the study used a treatment protocol designed based on the clinical experience of local experts, tailored specifically to the needs of the Asian population, and assessed in Korean subjects in real‐world clinical settings. This is especially important since Asian skin has unique physiological characteristics and aesthetic concerns [2, 13], but research on this population remains limited.

The findings of the study need to be interpreted with caution considering the limitations of the study, including the small sample size (*N* = 20), lack of a control group, and short follow‐up period (24 weeks). While the single‐arm, open‐label design provides valuable real‐world insights into treatment outcomes, the lack of a control group makes it challenging to rule out the possibility of placebo effects. Moreover, the injection protocol was specifically tailored for CPM‐HA20G to ensure optimal care. Hence, it may not be appropriate for use with a comparator group. Improvements were assessed primarily through pre‐ and post‐treatment comparisons. Considering the small sample size and the study's primary aim to explore key trends in clinical improvement with CPM‐HA20G in an Asian population under real‐world clinical practice, basic statistical methods were used to analyze data. This approach does not account for confounding factors, adjustments for multiple comparisons, and intra‐subject correlation across time points due to the longitudinal design. Considering these analytical constraints, we acknowledge the need for careful interpretation. As with other subjective assessments, GAIS is subject to potential bias. Incorporating independent assessors could have helped mitigate this bias to some extent, but this was not part of the study design. Furthermore, the generalizability of our study's findings is limited as we enrolled only Korean women aged 45 to 60 years. Therefore, the findings may not be broadly applicable to other Asian populations or diverse patient groups. The findings in this study should be further validated in future larger prospective trials or real‐world studies.

## 5 Conclusion

Our study provides preliminary real‐world insights into the effectiveness and safety of CPM‐HA20G in improving facial skin quality in an Asian population. Objective biophysical evaluations showed that skin quality parameters encompassing all EPCs significantly improved up to 24 weeks after treatment, with no serious adverse reactions noted during the study. Most subjects and investigators reported improvements in overall aesthetic appearance with the treatment. Recognizing the study's context and limitations, including the small sample size, lack of a control group, and analytical constraints, these findings should be interpreted with care and might not be entirely generalizable to all patients. Controlled trials with larger, more diverse patient profiles and longer study periods are needed to validate our preliminary findings and to confirm the long‐term effectiveness, durability, and safety of CPM‐HA20G in Asian populations.

## Author Contributions

**Je‐Young Park:** conceptualization, methodology, investigation, writing – original draft, writing – review and editing. **ChoonShik Youn:** conceptualization, methodology, investigation, writing – original draft, writing – review and editing. **Chanwoo Lee:** conceptualization, methodology, investigation, writing – original draft, writing – review and editing. **Kyou Chae Lee:** conceptualization, methodology, investigation, writing – original draft, writing – review and editing. **Heawon Shin:** conceptualization, methodology, investigation, writing – original draft, writing – review and editing. **Kkot Bora Yeom:** conceptualization, methodology, investigation, writing – original draft, writing – review and editing. **Juhyuk Park:** conceptualization, methodology, investigation, writing – original draft, writing – review and editing. **Sungkyu Jung:** conceptualization, methodology, investigation, writing – original draft, writing – review and editing. **Joo Ha Kim:** conceptualization, methodology, investigation, writing – original draft, writing – review and editing. **Wonkyu Hong:** conceptualization, methodology, investigation, writing – original draft, writing – review and editing.

## Ethics Statement

The study received approval from a central institutional review board (Human Co. Ltd. Skin Clinical Trial Center Institutional Bioethics Committee; IRB number: HM‐IRB‐P22‐0362; approved date: Jan 7, 2023). All subjects provided written informed consent before the study commenced. Additionally, subjects gave written informed consent for the publication of their photographs. The study was conducted in compliance with good clinical practice guidelines and applicable local regulations.

## Conflicts of Interest

Je‐Young Park: served on advisory boards and as a speaker for Merz Aesthetics. ChoonShik Youn, Chanwoo Lee, Kyou Chae Lee, Heawon Shin, Kkot Bora Yeom, Juhyuk Park, Sungkyu Jung, Joo Ha Kim: no relevant conflicts of interest to disclose. Wonkyu Hong: served on advisory boards for Merz Aesthetics Korea and Pierre‐Fabre Korea and received speaker honorarium from Merz Aesthetics Korea and Cynosure Korea.

## Supporting information


Appendix S1.

Figure S1.

## Data Availability

The data that support the findings of this study are available from the corresponding author upon reasonable request.
