# Supplementary material for: Facial Skin Quality Improvement After Treatment With CPM‐HA20G: Clinical Experience in Korea
Source: J Cosmet Dermatol. 2025 Jan 22;24(1):e16795. doi: 10.1111/jocd.16795 (PMC11755000; doi:10.1111/jocd.16795)
Supplement: Supplementary file 1 — Appendix S1. [file JOCD-24-e16795-s002.pdf]

A. Example of ‘very much improved’ rated by both subject and investigator at W12 and W24

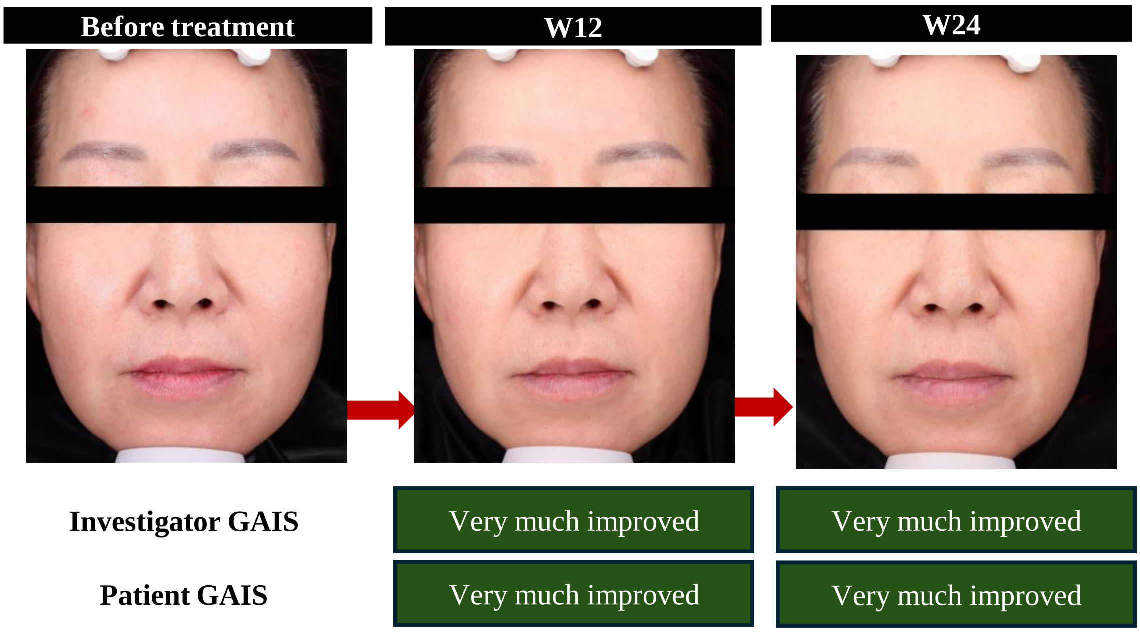

B. Example of ‘very much improved’ rated by both subject and investigator at W12 and ‘much improved‘ at W24

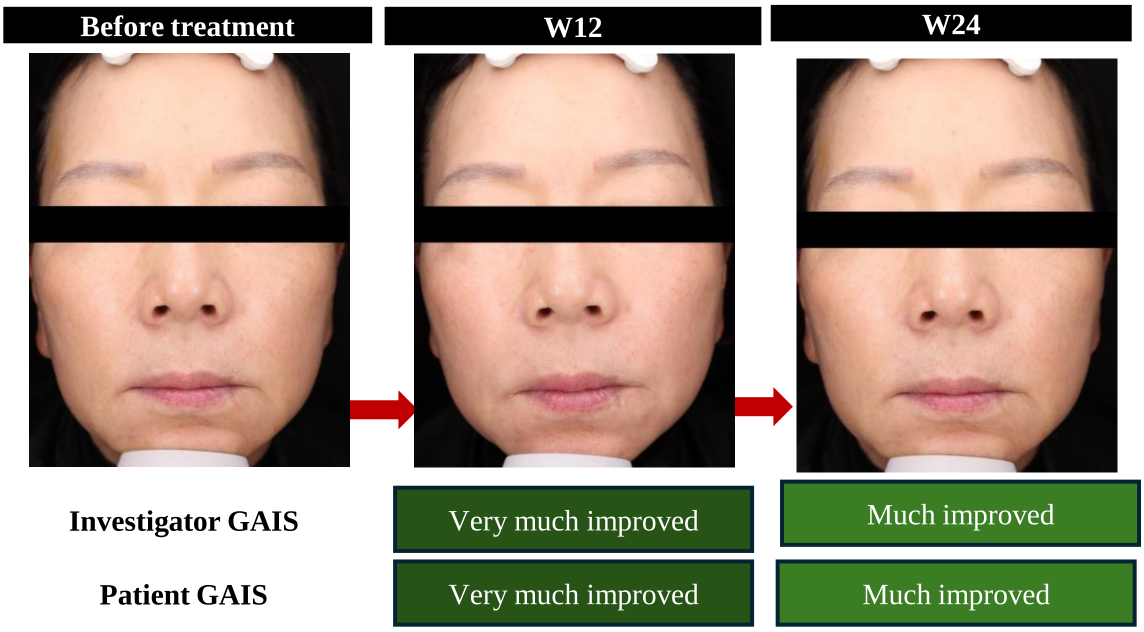

C. Example of ‘much improved’ rated by both subject and investigator at W12 and W24

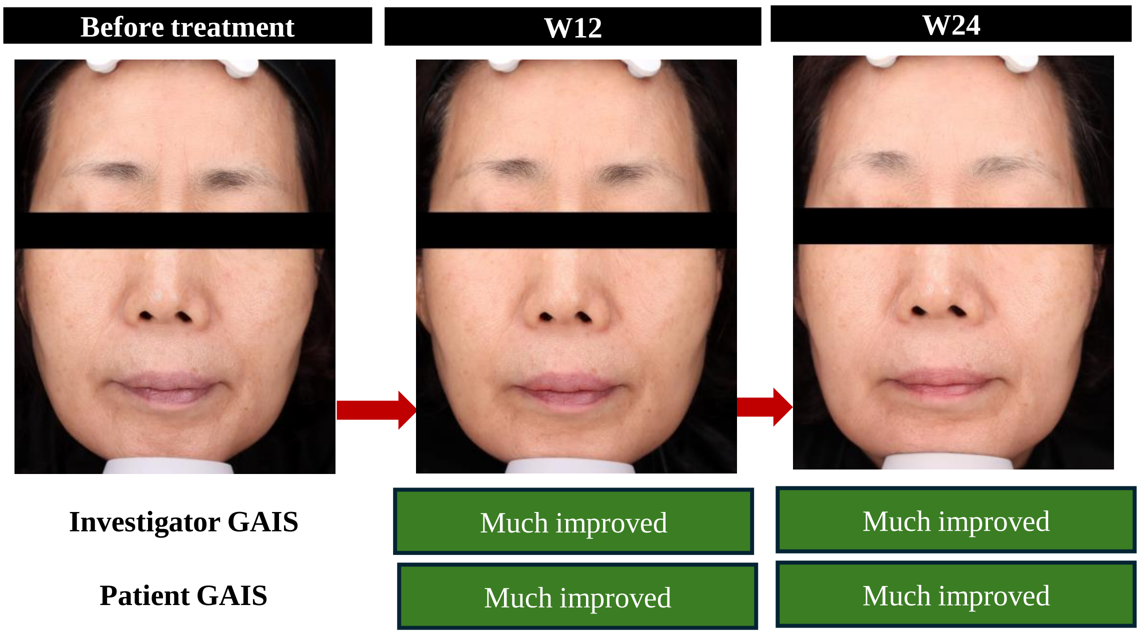

Supplementary Figure 1. Representative figures depicting different trend of overall aesthetic improvement at Week 12 and Week 24.

Abbreviations: GAIS, Global Aesthetic Improvement Scale; W, week
