# Supplementary material for: Facial Skin Quality Improvement After Treatment With CPM‐HA20G: Clinical Experience in Korea
Source: J Cosmet Dermatol. 2025 Jan 22;24(1):e16795. doi: 10.1111/jocd.16795 (PMC11755000; doi:10.1111/jocd.16795)
Supplement: Supplementary file 2 — Figure S1. [file JOCD-24-e16795-s001.docx]

**Supplementary Appendix 1. Complete list of eligibility criteria**

**Inclusion criteria**

1. Healthy adult women aged 45 to 60 years desiring improvement in skin quality.
2. Subjects voluntarily signed an agreement to participate in the study after receiving a sufficient explanation of the purpose and context of the study.
3. Healthy women without acute or chronic physical diseases, including skin diseases.
4. Available for follow-up during the study period.

**Exclusion criteria**

1. Subjects who were pregnant or had plans of becoming pregnant or breast-feeding.
2. Subjects who had psychiatric disease and infectious skin disease.
3. Subjects who had used an ointment containing steroids for more than 1 month.
4. Subjects who participated in a similar study within the past 6 months.
5. Subjects who had sensitive and hypersensitive skin (refer to individuals with active inflammatory dermatoses such as eczema and psoriasis).
6. Subjects who had skin disorders such as moles, pimples, red spots, scalds (burns), hemotelangiosis, and scars on the treatment areas.
7. Subjects who had used cosmetics or drugs on the treatment areas within the past 3 months.
8. Subjects who received treatment on the treatment areas from a dermatologist or aesthetic physician within the past 6 months.
9. Employees of the Human Skin Clinical Trial Center.
10. Subjects who were considered unsuitable by the investigator.
